# Bungling

**Published:** 1869-08

**Authors:** 


### BUNGLING.

How many of the honest laboring poor blindly bungle along through the world in misdirected efforts, and for no other reason apparently than that their fathers did it before them. It is positively painful to see day laborers working in a broiling sun at mid-day—masons, carpenters, hod-men, painters—the perspiration streaming from every pore, their faces sweltering with heat, and their whole appearance indicating that they were almost ready to faint. More work could be done with less wear and tear of the system, if breakfast weie taken at daylight, and work begun half an hour before sun-rise, knock off at twelve, sleep two hours, dine at two, go to work at three, and be in bed at nine. There is no necessary reason why some such plan as this should not be adopted, thus working in the cool of the day and resting during its fiercest heats; many a life would be saved by it every year and a large amount of sickness prevented.

